# Gain-of-Function Mutant p53: All the Roads Lead to Tumorigenesis

**DOI:** 10.3390/ijms20246197

**Published:** 2019-12-08

**Authors:** Yan Stein, Varda Rotter, Ronit Aloni-Grinstein

**Affiliations:** 1 Department of Molecular Cell Biology, Weizmann Institute of Science, Rehovot 7610001, Israel; yan.stein@weizmann.ac.il (Y.S.); ronitag@iibr.gov.il (R.A.-G.); 2 Department of Biochemistry and Molecular Genetics, Israel Institute for Biological Research, Ness-Ziona 7410001, Israel

**Keywords:** mutant p53, gain of function, tumorigenesis, p53 reactivation, p53 network

## Abstract

The p53 protein is mutated in about 50% of human cancers. Aside from losing the tumor-suppressive functions of the wild-type form, mutant p53 proteins often acquire inherent, novel oncogenic functions, a phenomenon termed mutant p53 gain-of-function (GOF). A growing body of evidence suggests that these pro-oncogenic functions of mutant p53 proteins are mediated by affecting the transcription of various genes, as well as by protein–protein interactions with transcription factors and other effectors. In the current review, we discuss the various GOF effects of mutant p53, and how it may serve as a central node in a network of genes and proteins, which, altogether, promote the tumorigenic process. Finally, we discuss mechanisms by which “Mother Nature” tries to abrogate the pro-oncogenic functions of mutant p53. Thus, we suggest that targeting mutant p53, via its reactivation to the wild-type form, may serve as a promising therapeutic strategy for many cancers that harbor mutant p53. Not only will this strategy abrogate mutant p53 GOF, but it will also restore WT p53 tumor-suppressive functions.

## 1. Introduction

The tumor suppressor p53 functions as the main regulator of several major signaling and cell-fate-decision pathways. Subsequent to various stresses such as DNA damage, oncogene activation or others, p53 undergoes post-translational modifications that lead to its activation, stabilization, and accumulation in the cell. The tumor-suppressor activity of p53 is mainly attributed to its transcriptional regulation of genes that are involved in numerous cellular processes, such as cell cycle arrest, apoptosis, senescence, DNA repair, and differentiation [1,2]. Moreover, accumulating data points to p53 involvement in numerous biological processes, such as forming a barrier to stem cell formation [3,4], metabolism [5,6], regeneration [7], prevention of liver pathologies [8,9], interaction with viruses [10], endocrinology circuits [11] and serving as the guardian of tissue hierarchy [12].

In nearly 50% of human cancers, p53 is found to be mutated, and in many of the remaining cases, where the WT allele is retained, other components in the p53 pathway are defected [13]. Mutations in most of the other tumor suppressors cause the loss of protein production, or the production of truncated or unstable proteins. In contrast, p53 is known to acquire missense mutations that lead to the production of a full-length protein, which tends to accumulate to high levels in tumor cells [14,15]. The most common missense mutations occur in six “hot-spot” amino-acids. Most of these mutations are found in the DNA-binding domain (DBD) of p53 and abrogate its transcriptional activity [16]. Generally, mutations in p53 are divided into two groups [17]. The first group consists of DNA contact mutations, such as p53^R248Q^ and p53^R273H^, which affect the domains of the protein that are directly involved in DNA binding. The second group consists of conformational mutations, such as p53^R175H^ and p53^H179R^, which cause either a full or partial distortion of the correct folding of the DBD of the p53 protein.

A unique feature of mutant p53 is its gain-of-function (GOF), which endows it with inherent oncogenic functions that are independent of the loss of WT p53 tumor-suppressor activity [18,19]. One of the first demonstrations of mutant p53 GOF was observed in *TP53*-null cells that were transfected with the over-expressing vector of mutant p53. Upon injections administered to mice, *TP53*-null cells formed local tumors that later regressed, while mutant p53 over-expressing cells formed lethal tumors [20]. Furthermore, mutant p53 knock-in mice show novel types of tumors and a significantly higher rate of metastases compared to p53−/− mice, thus demonstrating p53 GOF in vivo [21,22]. Various mechanisms were found to account for this GOF, including increased proliferation, inhibition of apoptosis, resistance to chemotherapy and enhanced inflammation, angiogenesis and invasiveness [18]. In this review, we describe some of the main roads by which GOF mutant p53 leads a cell towards tumorigenesis, and highlighted the broad interactions that mutant p53 has with numerous proteins, as well as its numerous transcriptional targets.

## 2. Disruption of Cell Cycle Control

WT p53 was shown to be involved in cell cycle regulation, promoting cell cycle arrest or cell death [23]. Thus, it is not surprising that mutant p53 was found to disrupt cell cycle control, leading to enhanced proliferation. Indeed, the notion that GOF p53 accelerates cell proliferation is well-established [24,25]. In studies aimed to understand the mechanism leading to accelerated proliferation, it was shown that tumor-derived p53 mutants interact physically with the master cell cycle regulator nuclear factor Y (NF-Y). These protein complexes can increase DNA synthesis in response to DNA damage through an aberrant upregulation of the expression of the NF-Y cell-cycle target genes, such as cyclin/*CDK1* kinase complexes [26]. It is worth mentioning that these genes are clustered with other cell cycle control genes and are annotated as a “proliferation cluster” [27]. In a following study, it was found that mutant p53 interacts with Yes-associated protein (YAP), and together they form a complex with NF-Y, that interacts with the regulatory regions of cyclin A, cyclin B, and *CDK1* genes [28]. This was further established in a genome-wide analysis showing that GOF p53 recognizes the promoters of genes encoding cyclin A (*CCNA2*), which is necessary for origin firing, and *CHK1*, which is required for preventing a collapse of replication forks, and transcriptionally activates their expression, in a cell-cycle-dependent manner, by localizing on their upstream regulatory sequences [29].

Mutant p53 was also found to trigger the activation of non-coding effectors, such as the circular RNA circPVT1 and miR-497-5p, leading to uncontrolled proliferation through the abnormal enhancement of the expression of cell-cycle-regulated genes. This is regulated through the mutant p53/YAP/TEAD complex via its regulatory region [30]. On the other hand, mutant p53 was shown to suppress the expression of miR-27a, resulting in augmented cell proliferation due to enhanced epidermal growth factor receptor (EGFR) signaling, resulting in the activation of the extracellular signal-regulated kinase (ERK) pathway [31]. In addition, various mutant p53 forms were shown to bind and activate STAT3, leading to increased invasion and tumor growth in colorectal cancer [32]. Aside from affecting signaling pathways, mutant p53 was shown to regulate different chromatin regulators, including the methyltransferases *MLL1* and *MLL2*, and the acetyltransferase *MOZ*. This regulation was shown to globally affect histone modifications, and to promote the proliferation of cancer cells [33]. Thus, it may be concluded that mutant p53 does not affect only cell signaling and specific gene transcription, but may also underlie global chromatin changes that occur in cancer cells, which facilitate their malignant phenotype.

## 3. Genomic Instability

Cells harboring mutant p53 exhibit extensive chromosomal aberrations. Mutant p53-expressing pre-tumor thymocytes, but not p53−/−cells, were shown to possess interchromosomal translocations. Moreover, after DNA damage, the G2–M checkpoint was found to be impaired in these p53-mutant cells. The mechanism behind this observation was attributed to the interactions of the p53 mutants with the nuclease Mre11, leading to less binding of the Mre11–Rad50–NBS1 (MRN) complex to DNA double-stranded breaks (DSBs), accounting for impaired Ataxia-telangiectasia mutated (ATM) activation [34]. Notably, genomic instability was also observed in mutant p53 transgenic mice [35,36,37].

Recently, a mechanistic link between mutant p53 expression and a cellular engulfment process, which leads to cell-in-cell (CIC) structures, was described [38]. Cell-in-cell (CIC) structures are mainly seen in aggressive tumors and are related to chromosomal structural abnormalities in human tumor tissue. The study concluded that cell ingestion in the presence of mutant p53 may drive entotic engulfment that would cause abnormal cellular division, and that mutant p53 would allow the abnormal progeny to survive. Interestingly, phosphorylated CHK1 (p-CHK1) was detected in the DNA of the engulfing cells. As mentioned above, *CHK1* is upregulated by mutant p53 and prevents the collapse of cells with origins of replication forks. A large-scale genomic study in a set of tumors led to the observation that there is an association between CIC and somatic copy number aberrations. Support for the observation that mutant p53 expression and CIC are linked to genomic instability was provided by the report that chromosomal instability was detected in the Pdx1-Cre; LSL-KrasG12D/+; LSL-Trp53R172H/+ mice, but not in their p53 null counterparts. Moreover, tripolar mitoses in CIC structures were often recorded in the mutant p53 mice and not in the p53 null mice [38].

## 4. Promoting Dedifferentiation and Acquiring Stemness Properties

The role of WT p53 in differentiation is well documented [39,40,41,42,43]. Moreover, WT p53 has been implicated in the regulation of stem cells self–renewal and differentiation and as a barrier to cancer stem cell (CSC) formation [3]. Thus, it was conceivable to foresee that GOF mutant p53 will contribute to the dedifferentiation processes and the formation of CSCs. Indeed, tumors harboring mutant p53 seem to possess an undifferentiated and aggressive phenotype. p53 mutations were shown to be restricted to poorly differentiated thyroid tumors and thyroid cancer cell lines [44]. Interestingly, examining different areas within a tumor revealed that the mutant p53 was confined to the less differentiated part of the tumor, presenting a positive correlation of mutant p53 and dedifferentiation [44] (for more examples of p53 aberration and dedifferentiation phenotypes, see Table 1 in [3]). Moreover, in breast and lung tumors, stem-cell-like patterns of transcription were found to coincide with the abolishment of WT p53 and the presence of p53 mutations [45]. These data are in line with the observation that WT p53 restrained the reprogramming process of mouse embryonic fibroblasts (MEFs) into induced pluripotent stem cells (iPSCs) [46,47,48,49,50], while GOF mutant p53 enhanced the reprogramming efficacy along with augmented tumorigenicity of the reprogrammed cells [51]. Moreover, accumulation of mutant p53 in progenitor-like cells in the subventricular zone-associated areas in the brain was shown to lead to the initiation of glioma, suggesting improper maturation of neural stem cells due to the presence of mutant p53 [52]. Similarly, mutant p53 bone-marrow mesenchymal stem cells are tumorigenic and induce sarcomas [53]. Interestingly, tumor cell lines derived from these sarcomas exhibited enhanced tumorigenic potential. These cells were smaller in size, had a circular morphology and high proliferation rate [54], characteristics that are assigned to iPSCs and stem cells [55,56]. Moreover, these cells highly expressed cancer- and CSC-related genes and an ESC gene signature. This signature correlates with the one seen in human tumors harboring p53 missense mutation, and is associated with poor patient survival. Furthermore, mutant p53 knockout led to a reduction in the tumor initiation capacity of these tumor-derived cell lines and in the expression of the ESC gene signature, supporting a link between mutant p53, stemness, and tumorigenesis. It is intriguing to speculate that specific genes identified in this ESC signature, can be viewed as a broader signature of CSCs and tumor progression toward a progressive disease, and may be used as biomarkers for disease progression. Recently published, mutant p53 was shown to promote aberrant self-renewal in acute myeloid leukemia (AML). This gain-of-function activity of mutant p53 is mediated by FOXH1, which can bind and regulate the expression of stem cell genes [57].

## 5. Modulation of Metabolism

It is well-established that cancer cells rewire a wide variety of metabolic processes to fulfill their energetic demands, as well as their anabolic needs, which require an excess of building blocks [58]. The roles of WT p53 in the regulation of metabolism are well-documented, including suppression of the Warburg effect [5,59,60], as well as controlling the response for glucose [61], lipid [6] and amino-acid starvation [62,63]. Mutant p53 was shown to modulate cancer cell metabolism in several ways. First, mutant p53 was shown to facilitate the cancer cell’s stress response to starvation due to the depletion of metabolites. Several mechanisms were shown by which mutant p53 ameliorates cell starvation, such as inhibition of the starvation response regulator AMP kinase (AMPK) [64], and amelioration of glutamine starvation response, primarily by activating canonical WT p53 targets—p21 in particular [65]. Aside from attenuating starvation response, mutant p53 is also implicated in the accumulation of metabolites, which are essential for tumor cells’ oncogenic properties. For example, in high-grade serous ovarian cancer (HGSOC), mutant p53 was shown to regulate the amount of lipids such as lysophosphatidic acid (LPA). This study shows that mutant p53 leads to LPA accumulation by down-regulating the LPA degrading enzyme, ACP6, in a manner that was sufficient to support HGSOC [66].

The second way by which mutant p53 may modulate cancer cell metabolism is by inducing the mevalonate pathway [67,68], which is implicated in a wide variety of physiological and pathological conditions, including cancer [69]. The targeting of the mevalonate pathway in cancer has been examined in different pre-clinical and clinical studies [69,70]. Intriguingly, it was demonstrated that mutant p53 and the mevalonate pathway exhibit reciprocal interplay, in which mutant p53 upregulates genes pertaining to the mevalonate pathway [67], while the mevalonate pathway was found to be a critical modulator of mutant p53 protein stability, but not the stability of the WT p53 protein [68,71,72,73]. Furthermore, statins, which inhibit a key enzyme in the mevalonate pathway, were shown to attenuate mutant p53 GOF effects, both by downregulating mevalonate pathway-related genes [67], as well as by reducing mutant p53 protein stability [68,71,72,73]. In all, these data indicate that not only it is possible to target mutant p53 harboring tumors by inhibiting metabolic changes induced by mutant p53, but it is also possible to target mutant p53 itself by using agents targeting metabolic pathways, such as statins.

A notable protein that was shown to mediate various metabolic effects of mutant p53 is NRF2. One of the metabolic effects that was shown to be mediated by NRF2 is the regulation of the response to oxidative stress. Although excessive levels of reactive oxygen species (ROS) are toxic to cells, moderately elevated ROS levels are beneficial for cancer cells, as they may activate a variety of signaling pathways that promote cell proliferation and survival [74]. Indeed, different studies indicate that tumor cells exhibit elevated levels of ROS and oxidative stress, which also correlate with tumor cells aggressiveness, as well as with poor prognosis in cancer patients [74,75,76,77,78]. In a previous study that was conducted in our lab, we reported that mutant p53 causes the increase of ROS by diminishing the expression of ROS detoxifying enzymes, via decreasing the induction of NRF2 [79]. A later study corroborated this observation, and demonstrated that the accumulation of ROS in mutant p53 cancer cells can be exploited as a therapeutic strategy, by pharmacologic depletion of glutathione (GSH), which specifically targets mutant-p53-expressing cells [80]. Protein homeostasis is another mutant p53 phenotype that was shown to be mediated by NRF2, through the regulation of proteasome activity [81]. The mutant p53–NRF2 interaction was shown to upregulate the expression of various proteasome subunit genes that lead to increased proteasome activity, which, in turn, lead to inhibition of tumor suppressors, such as KSRP [81]. Aside from NRF2, mutant p53 was shown to increase proteasome activity via upregulating the proteasome activator REGγ [82].

Another noteworthy metabolic effect mediated by mutant p53 is the enhancement of the Warburg effect by various mechanisms, including increased glucose intake via the GLUT1 transporter [83], as well as increasing glycolytic activity and decreasing mitochondrial oxidative phosphorylation [84]. Intriguingly, it was demonstrated that the R72 polymorphic form of mutant p53 endows cancer cells with enhanced oxidative-phosphorylation activity via the regulation of PGC-1α, thus leading to increased invasiveness and metastatic potential [85]. In that regard, it is interesting to note that in our model of highly aggressive, mutant p53 expressing CSC-like tumor lines [54], we observed several metabolic effects. These effects were, at least partially, mutant-p53-dependent, including increased glucose uptake, which correlates with increased GLUT1 membranal translocation, as well as increased mitochondrial mass and oxidative metabolism, as compared to the parental cells from which the tumor lines were derived [86]. The latter study, as well as the study linking mutant p53 forms with increased mitochondrial activity via PGC-1α, are in line with studies that reported increased expression of PGC-1α, as well as mitochondrial activity, in CSCs [87,88]. Taking the aforementioned findings together, it is tempting to speculate that mutant p53 might regulate metabolic changes depending on the cellular context, by suppressing mitochondrial metabolism in non-CSCs to promote the Warburg effect, and conversely, increasing mitochondrial metabolism in CSCs, to enhance their stemness.

## 6. Tumor Microenvironment

The microenvironment may help to block tumor formation, even when adjacent cells harbor potentially oncogenic genetic aberrations [89]. However, upon progression of the tumorigenic process, the microenvironment may change its phenotype and actively promote tumorigenesis [89]. We have recently published a review [90] summarizing the tumor–stroma crosstalk and the role of mutant p53 in this interaction, and proposed that the microenvironment may be modulated by mutant p53 to promote tumorigenesis. We suggest that mutant p53 may function as a key player in the modulation of the tumor–stroma crosstalk both at the initiation as well as the progression of the tumor–stroma vicious cycle, during which mutant p53 facilitates the adaptation (“re-education”) of the stroma cells to tumorigenic-promoting cells in the stromal compartment, as well as causing a modulation of the signals arriving from the stromal cells in a way that benefits the tumor cells.

## 7. Mother Nature Knows Best—Retrieving WT Function

The presence of a mutant p53 in various stem cells such as embryonic stem cells (ESCs), iPSCs or adult stem cells may lead to a lethal outcome. Mother Nature evolved various elegant mechanisms to detain these fatal consequences. Indeed, despite malignant properties of mutant p53 in somatic cells, mutant p53 mice develop into viable embryos and mature organisms, and develop aggressive tumors only in adulthood. This phenomenon is intriguing, especially in light of the extreme sensitivity of ESCs to DNA damage and the need for an active WT p53 to protect these cells, suggesting a mechanism to restrain mutant p53 function. A comprehensive study [91] has revealed that WT and mutant p53 ESCs exhibit similar characteristics in doubling time and in vitro differentiation potential, and that despite the difference in p53 status, both ESCs remained pluripotent in vivo and gave rise to benign teratomas upon injection into nude mice. Moreover, mutant p53 ESCs displayed only minor karyotype changes, pointing to an active tumor-suppressive function within these mutant ESCs. Strikingly, analysis of the p53 protein conformation within the mutant ESCs revealed a WT conformation in a ratio of approximately 1:1 with the mutant conformation. This conformational change allowed the activation of DNA damage-responsive genes in these mutant ESCs, in a similar pattern as the one obtained in WT p53 ESCs, providing genomic stability to the mutant ESCs and the birth of mice with no malignant signature.

Another mechanism to maintain at least one WT allele is the loss of heterozygosity (LOH). We have shown that the classical well-known WT p53 LOH is correlated with genome stability of the cells: higher frequencies of LOH were observed in somatic cells compared to adult stem cells, in an age-dependent manner, while LOH was greatly attenuated in iPSCs and even more in ESCs [53]. Interestingly, it was found in single-cell-subclones of iPSCs and MSCs that LOH is a bi-directional process, namely, not just the WT allele may be lost, but events of loss of the mutant allele were also recorded. Strikingly, the majority of the LOH events in ex vivo bone marrow progenitors led to the loss of the mutant allele, suggesting a mechanism that dictates a preference to retain the WT allele and to exclude the mutant allele, assuring genome stability in vivo.

Turrell et al. [92] presented data of yet another mechanism by which the cell may protect itself against the tumorigenic effect of mutant p53. Their data argue against a dominant-negative effect of mutant p53 on the WT form in lung tumors, but rather suggest a dominant-positive function of the WT p53 on the mutant form. Even in the presence of mutant p53, WT p53 retained its transcriptional activity towards anti-proliferative and apoptotic responses [92]. This observation can be explained either by the fact that p53 mutant:WT heterotetramers may retain, in these cells, some WT functions, or that there is a bias towards WT p53 homotetramer formation. Indeed, a subsequent study [93] revealed that WT p53 oligomerization is more efficient than the oligomerization of mutant p53. At equimolar levels of WT p53 and mutant p53, mutant p53 GOF activities are restrained. This may explain the need for the accumulation of mutant p53 in tumors. High levels of mutant p53 contribute not just to the dominant-negative functions of mutant p53, but also to the abolishment of dominant-positive functions of the WT form. Finally, yet importantly, the mechanism by which Mother Nature protects large animals, such as elephants, from cancer, is worth mentioning. It is believed that elephants have a lower-than-expected rate of cancer, when compared to other mammals, due to their multiple copies of *TP53*, which expanded, coincidently with the evolution of large body size. As a result, elephant cells demonstrate increased apoptotic response following DNA damage, compared to human cells [94,95].

## 8. Concluding Remarks

WT p53 functions as a major regulator in many cell-fate-determining processes. It executes its guardian missions through various interactions with other proteins and by transcriptional regulation of different genes. Interestingly, mutations in the WT form not only lead to the abrogation of its normal functions, but also give rise to an active antithetical protein, with its own “social network” of interacting proteins and transcriptional targets that endows it with GOF activities (Table 1). Since the formation of these mutants of p53 is fatal to cells, Mother Nature has evolved mechanisms such as LOH and conformational “correction” of the mutant conformation to secure the cells. This “re-education” of mutant p53, namely, the various mechanisms by which Mother Nature attempts to ameliorate the effects of mutant p53, not just abrogates the GOF of the mutant form, but also reinstates WT p53 tumor-suppressive functions. Based on the wisdom of Mother Nature, the development of new cancer medicines, aimed at the correction of the mutant p53 form [96,97], may provide new horizons in cancer treatment.

## Figures and Tables

**Table 1:** “Social network” of mutant p53 ^1^.

Cancer Hallmarks Associated with GOF		Mutant p53 Interacting Molecules/Gene Targets	Ref.
**Proliferation/Abrogation of Growth Suppressors**	Protein interactions (*indirect)	**NF-Y** (R175H, R273H), **p300** (R175H, R273H, R280K), **TopBP1** (V143A, R175H, R248W, R249S, R273H), **MCM4 and PCNA** (R273H), **YAP** (R175H, H193L, L194F, R273H) TEAD* (R175H, H193L), **EGFR** * (R273H), **ETS2** (R175H, R248Q, R248W, R273H), **STAT3** (R175H, R248W, R248Q, R273H, R282W)	[25,26,28,30,31,32,33,82,98,99]
	Transcriptional targets (*indirect)	***REGγ*** (R175H, R248W, R273H, R282W), **nucleotide metabolism genes** (**NMGs**) (R175H, R249S, R273L, R273H, R280K), ***Axl* receptor tyrosine kina*se*** (R175H, R267P, R273C, R273H, D281G), **cyclin A+cyclin B2+*CDK1*** (R175H, H193L, R248L, R273H, R280K), **circPVT1** (R175H, H193L), **miR-27a** (R273H), ***MLL1***, ***MLL2* and *MOZ* chromatin regulators** (R248Q, R248W, R249S, R273H)	[28,30,31,33,82,100,101]
**Resistance to Cell Death/Chemoresistance**	Protein interactions (*indirect)	**NF-Y** (R273H, R249S), **ZEB1** (R273H), **p300** (R273H), **p73** (R175H, R248W, R273H, R273C), **VDR** (R175H, R280K), **ETS1** (D281G), **ETS2** (R175H, R248W), **NRF2** (R175H, R280K), **PELP1** (R273H,280K), **Caspase 3** (R175H, R273H) **Caspase 9** (D42Y, R175H, R337H), **FOXO1** (R175H, D281G)	[79,102,103,104,105,106,107,108,109,110,111,112,113]
	Transcriptional targets (*indirect)	***EFNB2*** (R273H), ***SLC25A1*** (R175H, G245A, R273H, R280K, D281G), **miR-223** (Negative regulator, R175H, R273H, R280K), ***STMN1*** (R273H, R280K), ***REGγ*** (R282W, R175H, R248W, R273H), ***KLF17*** (R175H, R280K, R273H, R282W), ***NF-κB2*** (R175H, R273H, D281G), **Bcl-xL** (R273H), **miR-128-2** (R175H), ***MDR1*** (V143A, R175H, R248W, R273H, D281G), ***ATF3*** (Negative regulator, R175H, H179G, R248W, D281G), ***MEF2D*** (Negative regulator, R175H), **dUTPase** (R175H, R248W), ***CD95/Fas*** (R175 H, R248W, R273H)	[102,112,113,114,115,116,117,118,119,120,121]
**Tumor Microenvironment/inflammation**	Protein interactions (*indirect)	**NF-κB** (R175H, R248Q, R273H), **DAB2IP** (R175H, M237I, R248W, R273H, R280K), **c-MAFF** (R273H), **BTG2** (Negative regulator, R175H, H179R)	[122,123,124,125]
	Transcriptional targets (*indirect)	**s*IL-1Rα*** (Negative regulator, R175H, R273H), ***CCL2*** (R248L), ***SDF1/CXCL12*** (murine R172H, R175H, R273H), **CXC chemokines** (R175H, H179L, R248Q, R273H, D281G) ***MMPs* and *IL-1β*** (R248Q, R273H), ***TGFβ-R2*** (Negative regulator, R175H, H179R, R248W), ***SOCS1*** (R175H, R248Q)	[124,125,126,127,128,129,130,131]
	**Exosome release**	**miR-1246** (V157F, R175H, R248W, R249S, R273H)	[132]
**Self-Renewal/Stemness**	**Transcriptional targets**	***FOXH1*** (mechanism unknown, murine R172H), **lnc273–31** and **lnc273–34** (R273H), **embryonic stem cell gene signature** (murine R172H), ***CD44*** (R248W, R273H) ***Lgr5*** (R172H, R273H) ***ALDH1A1*** (R175H R248W, R273H)	[45,54,57,133,134]
**Metabolism**	Protein interactions (*indirect)	**AMPKα** (R175H, P151S, R175H, G245C, and R282W), RhoA * (mechanism unknown, R175H, L194F, M237I, R280K), **SREBP proteins** (R273H), GAPDH* (R273H), **PGC-1α** (P72-mut-R175H and R273H), **NRF2** (R175H, M237I, R248Q, R249S, R248W, R273H, C277F, R280K)	[64,66,67,79,80,81,83,85,107,135]
	Transcriptional targets (*indirect)	***SLC25A1*** (R175H, G281D, G245A), ***SLC7A11*** (Negative regulator, R175H, G266E, R273H, C277F), ***HMOX-1*** and ***NQO-1*** (Negative regulators, R273H), *TXN* and ***PSMC-1*** (R175H, R280K), **mevalonate pathway enzymes** (R273H), **mitochondrial metabolism genes** (murine R172H), ***ACP6*** (Negative regulator, R175H, R249S and R273H), ***CDKN1A*** (**p21**) (R248Q, R273H), **Nucleotide metabolism genes** (NMGs) (R175H, R249S, R273L, R273H, R280K), **Proteasome subunit genes** (R175H, M237I, R249S, R273H, R280K)	[65,66,67,79,80,81,86,100,107,110]
**Genomic Instability**	Protein interactions (*indirect)	**E2F4** (R175H), **Mre11** (R248W, R273H)	[34,136]
	Transcriptional targets (*indirect)	***BRCA1*** (R175H), ***RAD17*** (R175H)	[136]
**Invasion and Metastasis**	Protein interactions (*indirect)	**p63** (V143A, R175H, H179Y, Y220C, I254R, R273H, R280K), **p73** (V143A, R175P, R175H, H179Y, Y220C, R248W, I254R, R273H, R280K), **PTEN** (R273H), **SMAD3** (C135Y, V143A, R175H, R248W, R273H, R282W), **NF-Y** (R175H, R273H), **SREBPs** (R273H), **Pontin** (R175H, R248Q, R273H) **KLF17** (R280K, R282W), RCP* and MET* (R175H, R248W, R273H) Integrins* (R175H, R273H), *ATF3* (V143A, R175H, R249S, R273H), ***MDM2*** (R175H, I254R, R273H), **SP1** (R248W, R280K), **NRD1** (R273H), **Pin1** (murine R172H, R175H, R280K), **STAT3** (R175H, R248W, R248Q, R273H, R282W)	[32,67,112,114,121,137,138,139,140,141,142,143,144,145,146,147]
	Transcriptional targets (*indirect)	***REGγ*** (R248Q), ***let-7i*** (S241F, R248W, P273H, R280K), ***KLF17*** (R280K, R282W), ***MYO10*** (R175H, R273H, R280K), STMN1* (R175H), ***PDGFR-β*** (R175H, Y220C, C242R, T155P, R248W, R273H, R280K), **Nucleotide metabolism genes (NMGs)** (R175H, R249S, R273L, R273H, R280K), ***DICER1*** (R273H, R280K), ***EFNB2*** (R175H, R248W, R273H, R280K), MYC* (R273H), ZNF652* and miR-155* (R248Q, R249S, R282W), FOXM1* (R175H, C238F, G245D, R248W), ***Notch3*** **and** CCNG1* (R248Q), Twist1* (epigenetic, R175H, R273H, P223L/V274F), *DLX2* and NRP2* (R273H), **endoplasmic reticulum UDPase *ENTPD5*** (C176S, R248W, R273H, R280K)	[100,112,114,121,137,140,145,148,149,150,151,152,153,154,155,156,157]

^1^ Genes/proteins that are part of mutant p53 network are marked in bold. Shown in parentheses are the mutant p53 forms that were implicated in the interaction or transcriptional regulation of the indicated proteins or genes, respectively.

## Abbreviations

AML	Acute myeloid leukemia
CIC	Cell-in-cell
CSC	Cancer stem cell
DBD	DNA binding domain
DSB	Double-strand breaks
ESC	Embryonic stem cell
GOF	Gain-of-function
HGSOC	High-grade serous ovarian cancer
iPSC	Induced pluripotent stem cell
LOH	Loss of heterozygosity
LPA	lysophosphatidic acid
LSL	Lox-Stop-Lox
MEF	Mouse embryonic fibroblast
MSC	Mesenchymal stem cell
NMG	Nucleotide metabolism gene
ROS	Reactive oxygen species
WT	Wild-type
